# XTMS: pathway design in an eXTended metabolic space

**DOI:** 10.1093/nar/gku362

**Published:** 2014-05-03

**Authors:** Pablo Carbonell, Pierre Parutto, Joan Herisson, Shashi Bhushan Pandit, Jean-Loup Faulon

**Affiliations:** 1 University of Evry, iSSB, F-91000 Evry, France; 2 CNRS, iSSB, F-91000 Evry, France; 3 IISER Mohali, SAS Nagar, Manauli, PO 140306, Mohali, Punjab, India

## Abstract

As metabolic engineering and synthetic biology progress toward reaching the goal of a more sustainable use of biological resources, the need of increasing the number of value-added chemicals that can be produced in industrial organisms becomes more imperative. Exploring, however, the vast possibility of pathways amenable to engineering through heterologous genes expression in a chassis organism is complex and unattainable manually. Here, we present XTMS, a web-based pathway analysis platform available at http://xtms.issb.genopole.fr, which provides full access to the set of pathways that can be imported into a chassis organism such as *Escherichia coli* through the application of an Extended Metabolic Space modeling framework. The XTMS approach consists on determining the set of biochemical transformations that can potentially be processed *in vivo* as modeled by molecular signatures, a specific coding system for derivation of reaction rules for metabolic reactions and enumeration of all the corresponding substrates and products. Most promising routes are described in terms of metabolite exchange, maximum allowable pathway yield, toxicity and enzyme efficiency. By answering such critical design points, XTMS not only paves the road toward the rationalization of metabolic engineering, but also opens new processing possibilities for non-natural metabolites and novel enzymatic transformations.

## INTRODUCTION

Synthesis of value-added molecules in chassis organisms, such as *Escherichia coli* and *Saccharomyces cerevisiae*, through heterologous pathways requires of a careful selection and optimization of each step of the biosynthesis pathway (1, 2). Successful examples of bioproduction of pharmaceutical products in microorganisms include the production of semisynthetic artemisinin (3), taxadiene (4), farnesol (5) or naringenin (6). To rationalize the engineering process of metabolic pathway design, retrosynthetic design approaches have been recently proposed (7–9). Retrosynthesis, a technique originally developed for synthetic chemistry, consists of searching routes producing a target compound by means of the reverse application of chemical transformations to the target compound and its precursors. In metabolic engineering, retrosynthesis is applied in order to perform an efficient search of potential pathways through the use of algorithms that iteratively import biosynthetic modules into a chassis organism until a suitable set of precursors is found. This approach was for instance used in the BNICE framework (9) by the application of generalized reaction rules that are described by the bonds that are either broken or created by the transformation, a reaction representation that can potentially lead to a big combinatorial set of solutions. The power of computer-assisted retrosynthesis for scoping the metabolic space and enumerating pathways relies, therefore, on the way chemical biotransformations are represented and describe important chemical features. In order to fully exploit the potential capabilities that are present in metabolic networks through retrosynthesis, our group has recently proposed a molecular signatures approach that codes for changes in atom bonding environments where the reaction is taking place (7). The advantage of such fingerprint method is that while the reaction rules describe the changes in the environments of the atoms belonging to the catalytic center of the reactions, the size of the atomic environment (named diameter *d*) can be tuned to control the combinatorial explosion of possible compounds.

The degree of plasticity in metabolic networks that is uncovered by the signatures representation reveals an intrinsic feature of organisms linked to their adaptability, i.e. enzyme promiscuity (10). Promiscuity stands for the ability of enzymes to catalyze more than one reaction or to accept more than one substrate, a mechanism that can be traced to the evolutionary origins of enzymatic functions (11, 12). Mimicking nature, such enzyme versatility can provide novel ways for synthetic biology to engineer circuits with the ability of processing chemicals associated with industrial, health or environmental applications (13, 14). In order to explore such enzyme capabilities for pathway design, present metabolic, enzyme and reaction databases like MetaCyc (15), KEGG (16), BRENDA (17), CHEBi (18) and RHEA (19) can provide a wealth of information. Online tools, such as PathPred (20), can predict plausible pathways both for biodegradation and biosynthesis of secondary metabolites from a set of reaction rules based on the KEGG RPAIR database (based on RDM patterns) (21). The authors proposed a score for each pathway as the average of the individual scores of each reaction given by the similarity between the query compound atoms and the matched compound atoms in the RDM patterns, which are a collection of chemical structure transformations generated for each reaction. However, RDM reaction rules are limited to biosynthesis of secondary metabolites and aspects such as toxicity or yields are not taken into account. Metabolic Tinker (22), in turn, is a web server for designing pathways between two given compounds. Metabolic tinker is based on the CHEBi and RHEA databases for pathway feasibility estimation by using thermodynamics. However, a main limitation in Metabolic Tinker is the fact that the user needs to provide the source and the target, which imposes a practical limit on the number of pathways that can be explored online. Another limitation of Metabolic Tinker is that the pathway search is not based on reactions rules but on a graph search of connected compounds expanded by means of a compound similarity threshold. Another similar tool is From Metabolite to Metabolite (FMM) (23), although it does not exploit full pathway information, such as thermodynamics.

Here we introduce the XTMS web server, an online tool which provides access to the set of available pathways that can be imported into *E. coli* based on an Extended Metabolic Space, i.e. on the set of potential *in vivo* biochemical transformations as modeled by molecular signatures (24). It differs from previous tools for pathway design (e.g. PathPred, Metabolic Tinker) by extending the pathway search to the Extended Metabolic Space and by providing a pathway ranking that considers context information, such as maximum allowable pathway yield and toxicity. In addition, a unique and useful feature of XTMS is that it performs a search on the enzyme sequence space to provide the list of top ranked pathway constructs.

## MATERIALS AND METHODS

### Data sources

**Figure 1.**
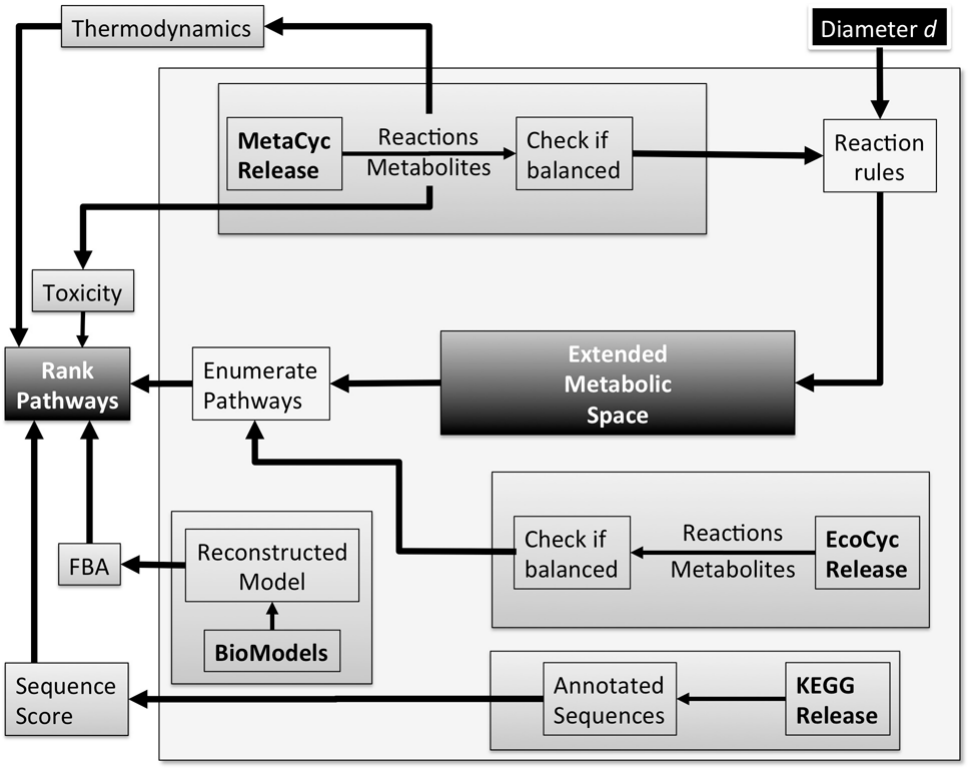
XTMS server curation process. (i) Metabolic reactions and metabolites are collected from the MetaCyc release; (ii) Data in MetaCyc is filtered in order to keep only balanced reactions; (iii) Metabolic reactions and metabolites are collected from the Ecocyc release; (iv) Data in EcoCyc is filtered in order to keep only balanced reactions; (v) The Retropath algorithm is applied in order to compute pathways producing heterologous compounds; (vi) Sequences encoding enzymes in the pathways are taken from the KEGG release and scored; (vii) Estimation of Gibss-free energies for the reactions is obtained from MetaCyc; (viii) Toxicity of metabolites is obtained from the EcoliTox server.

At present, data sources of XTMS are the following: (i) Reactions and metabolites: MetaCyc version 16.0; (ii) Metabolism of chassis organism: EcoCyc version 16.1; (iii) Gibbs energies: MetaCyc version 17.5; (iv) *E. coli* model for flux balance simulations: MODEL3023609334 (25) from BioModels database (26); (v) Enzyme sequence annotations: KEGG release 50. The curation process is detailed in Figure 1. Statistics of XTMS and third-party databases are provided in the server.

### Computation of the Extended Metabolic Space

The basic steps that allow determining and ranking in a given metabolic space those heterologous enzymatic steps producing a target compound from a chassis organisms have been previously defined by the authors (27). These basic steps are the following: (i) Define input, output and metabolic space; (ii) Define specifications of the desired circuit; (iii) Compute scope connecting input with output sets; (iv) Enumerate the circuit design space; and (v) Explore the design space in order to find the optimal solution(s). We have also described elsewhere the computational process that allows extending the metabolic space by proposing a solution based on molecular signatures (24). A chemical compound is coded into a molecular signature by collecting the information about how each individual atom connects to its neighbors through chemical bonds up to a predetermined distance (diameter) *d*. Reaction signatures (also named reaction rules) are computed as the net difference between the signatures of the products and of the substrates. The advantage of the signatures method is that the reaction rules describe the changes in the environments of the atoms belonging to the catalytic center of the reactions, whereas any group of atoms that remains essentially unchanged between substrates and products will not appear in the signatures. As the diameter *d* increases, the signature coding system becomes more specific and the size of the environment can be tuned to avoid a combinatorial explosion of possible compounds. The process of computing the Extended Metabolic Space for a given diameter *d* is computationally expensive and consists on the application of the reaction rules to all combinations of known metabolites so that the original set of metabolic reactions is extended with those additional putative promiscuous reactions that verify the rules at the given diameter *d*. The XTMS server implements the Extended Metabolic Spaces for diameters *d* = 14, 12 and 10, providing in that way a good trade-off solution between the information already available in metabolic databases and the ability of generalizing such information by including predicted promiscuous reactions that can be expected to be processed by those enzymes present in the network. The resulting databases are downloadable from the server.

### Input query and workflow

**Figure 2.**
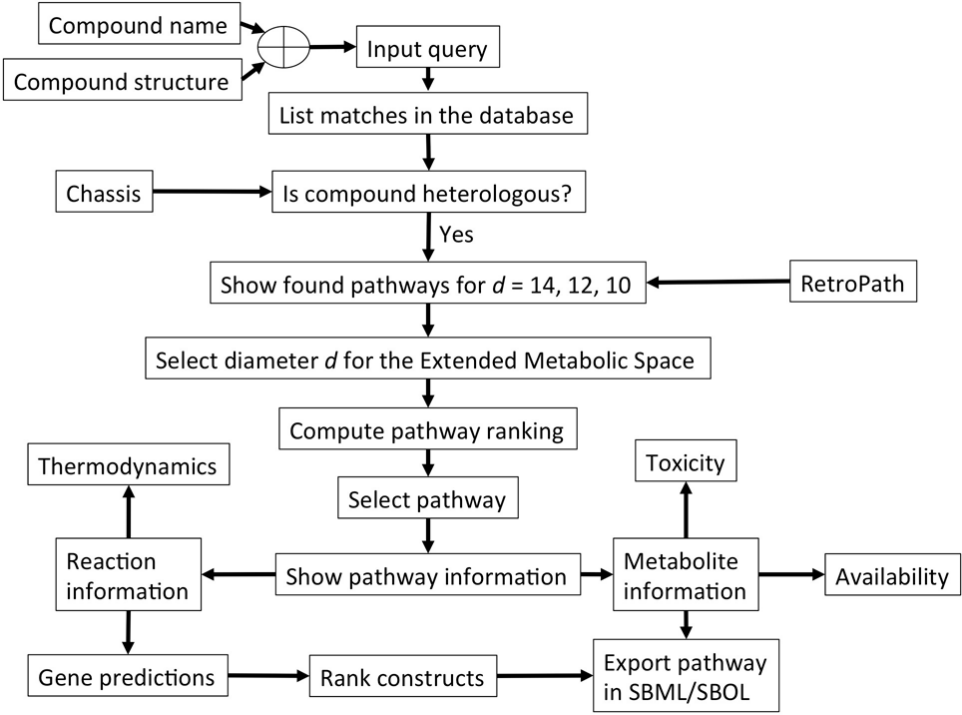
Schematic representation of the query process in the XTMS server. After the user inputs a query compound, pathways for heterologous compounds that match the query are retrieved from the Extended Metabolic Space by RetroPath. After selecting a desired diameter, pathways and constructs are ranked and information about reactions and metabolites is provided, with the possibility of downloading the desired construct in SBML/SBOL format.

The workflow of the XTMS server is shown in Figure 2. The query process starts when the user submits a compound that is non-naturally produced in the chassis organism *E. coli*. To that end, XTMS accepts as an input query either a molecular structure or a compound name. In the case of molecular structures, they can be both entered in MDL Molfile format or directly drawn through a chemical draw interface (JSDraw, Scilligence). Once the query is submitted, the user is presented with a list of compounds that corresponds to the matches in decreasing order of the query compound with chemicals that can be connected to the chassis in the Extended Metabolic Space. Matches for molecular structures are based on the Tanimoto similarity (28) search implemented in OpenBabel (29) between query structure and the compounds in XTMS, i.e. based on the ratio between the number of common substructures for each pair of compounds. For each match, the system informs if it is either endogenous or heterologous to the chassis organism (*E. coli*). The former are naturally produced by *E. coli*, while for the latter, the number of available pathways at diameters *d* = 14, 12 and 10 is given and the user can select to retrieve the list of pathways at the desired diameter.

The lists of pathways are obtained by running our RetroPath algorithm (27) for Extended Metabolic Spaces of diameters *d* = 14, 12 and 10. The workflow of RetroPath that finds and enumerates the pathways consists of (i) Computing the scope: the algorithm performs a two-step search, first a forward search to determine all compounds that are reachable, and then a backward step in order to add to the scope starting from the target compound any reaction that can be involved in a target-producing pathway imported into the chassis; (ii) Construction of the stoichiometry matrix corresponding to the target scope starting from precursors that are endogenous to the chassis; (iii) Apply elementary flux modes (30) in order to determine all target-producing pathways.

### Pathway ranking

Once a target compound and a diameter have been selected, XTMS will rank the enumerated pathways based on several criteria (7) and will generate a summary table with the ranked list of pathways and the following pathway information: (i) *Total score*: This is the total score of the pathway, which is computed by the weighted sum of the terms corresponding to gene score, toxicity, yield and Gibbs energy (see definitions below). Weights are chosen in order to normalize the final score; (ii) *Gene score*: The goal of this score is to provide an estimate of the performance of the pathway based on the combination of individual scores for the enzymes that putatively can catalyze each of the reaction steps *r*. The predictions are based on enzyme promiscuity through the tensor product technique (11, 31). The gene score is given by the average of each individual score; (iii) *Number of steps*: Number of enzymatic steps in the pathway; (iv) *Number of putative steps*: Number of enzymatic steps in the pathway that correspond to putative reactions predicted in the Extended Metabolic Space; (v) *Estimated toxicity*: Average toxicity log(IC50) for intermediate metabolites in the pathway, evaluated by using the EcoliTox server (32); (vi) *Maximum allowed yield*: This value estimates the maximum available yield in the pathway based on estimated fluxes of the precursors of the pathway in a wild-type strain of *E. coli* obtained by flux balance analysis optimized for growth. The value is computed as the minimum yield; (vii) *Estimated free Gibbs energy*: Standard Δ*G* Gibbs-free energy for reactions in kcal/mol were taken from MetaCyc database (15), where values are estimated by using the group contribution method (9); (viii) *Number of unfavorable reactions*: Number of reactions in the pathway that are not thermodynamically favorable (Δ*G* > 0), according to their estimated Gibbs-free energy.

### Pathway information

For the selected pathway, XTMS provides an interactive graphical depiction of the enzymatic steps, where the user can access reaction and compound information. In addition, four tables are given that correspond to (i) Reactions: list of reactions with associated Gibbs-free energy and EC (Enzyme Commission) number; (ii) Metabolite exchange: list of metabolites involved in the pathway with their net stoichiometric balance and predicted toxicity; (iii) Pathway yield: maximum allowable yield from precursors and main bottlenecks, i.e. precursors of the pathway that are also consumed for biomass production; (iv) Top constructs: list of 10 top constructs that can be obtained by combinations of predicted genes, based on the list of genes and individual scores for each pathway step. Each construct is downloadable as a file in SBML format (33) containing the pathway information with construct information annotated in SBOL format (34), facilitating in that way information exchange with programs that process biochemical models and DNA components.

### Reaction information

For each individual reaction in the Extended Metabolic Space, XTMS provides information about products and substrates and top ranked genes that are predicted to correspond to enzymes catalyzing the reaction, with associated scores.

### Implementation

The server runs under apache on a Linux machine running Ubuntu. The server was written in python by using the Django framework in combination with SQLite 3. It uses MetaCyc version 16.0 (15) and enzyme sequences were taken from the KEGG database (16). Chemical format interconversions were computed by using Openbabel (29). The RetroPath algorithm (27) was written in Perl for generation of the Extended Metabolic Space and in C with calls to the efmtool (35) for pathway enumeration.

## RESULTS AND DISCUSSION

A better understanding of the metabolic space and particularly the scope of metabolites that can be synthesized and transformed by enzymes is critical for future development in metabolic engineering and synthetic biology. To that end, the XTMS web server explores the regions in the extended metabolic space that can be reached through metabolic engineering. The computational procedure available at the web server provides a good balance between combinatorial complexity and predictive power from promiscuous reactions. For instance, the extended metabolic space that XTMS provides for diameter *d* = 12 was generated starting from a set of reaction rules and known metabolites from the MetaCyc database that comprised 6093 metabolites connected through 6078 reactions. After running the algorithm that performs the metabolic extension for 50 iterations, it converged to a total of 27 743 reactions, i.e. almost five times larger than the size of the initial network.

A valuable result from XTMS is the determination of the extent of the chemical space that can be reached through biochemical transformations from the chassis organism. Based on the application of the retrosynthesis principles for scope determination (7) and pathway enumeration by using elementary modes (36), we found 2182 heterologous compounds in the database that could be connected through metabolic pathways to the *E. coli* chassis. Concerning pathway enumeration, the number of pathways can substantially change from one diameter of the Extended Metabolic Space to another one. As the diameter *d* decreases, more pathways can be considered through new reactions. A case that illustrates this situation is the production of glycocholate (glycocholic acid). At diameter *d* = 14, only one pathway was found, while at decreasing diameters to *d* = 12 and *d* = 10, one additional pathway was found in each case. Therefore, the user should decide the degree of specificity in the reactions contained in the pathways, especially when dealing with cases where a large number of pathways can be formed (in more than 10% of the cases, we observed that number of available pathways at low diameters in XTMS is in excess of 100). Such large numbers require of appropriate techniques to rank pathways, so that optimal solutions can be selected for implementation. To that end, XTMS implements a pathway objective function as described in Materials and Methods. The selection of optimal production circuits is facilitated in that way.

**Figure 3.**
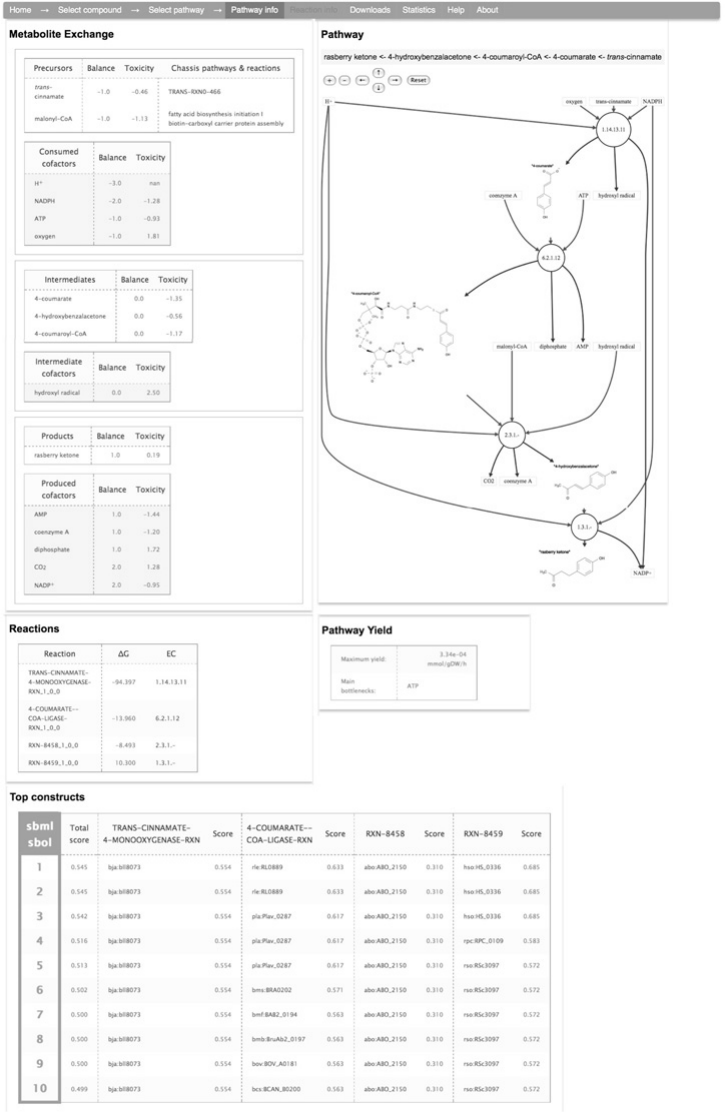
Example of pathway information page generated by XTMS. An interactive graphical depiction of the pathway is rendered along with four tables corresponding to reaction information, metabolite exchange information, pathway yield and list of top 10 predicted constructs.

As an example, Figure 3 shows the output of XTMS regarding pathway information for producing raspberry ketone [4-(4-hydroxyphenyl)-butan-2-one] at diameter *d* = 14. Raspberry ketone, which is found in some fruits, vegetables, berries and tree barks, is one of the most expensive flavor components used in the food industry (up to $20 000/kg may be paid) (37). Raspberry ketone is obtained in the raspberry (*Rubus idaeus*) by condensation of coumaroyl-CoA with malonyl-CoA (BAS enzyme) to form *p*-hydroxybenzalacetone, which is further reduced into raspberry ketone (BAR enzyme). For this example, the XTMS identified the two-step natural route of raspberry ketone production that starts from coumaroyl-coA, a compound that is found in many plants tissues as intermediate of the lignin biosynthetic pathway. Coumaroyl-coA, however, is not readily available in *E. coli*, and thus XTMS proposed the insertion of 4-coumarate ligase (4CL enzyme) to produce it from 4-coumarate. In order to obtain this metabolite, three alternative heterologous pathways are proposed: (i) cinnamate 4-hydroxylase (EC 1.14.13.11); (ii) tyrosine ammonia lyase (EC 4.3.1.5); (iii) the two-step conversion hydroxyphenylpyruvate reductase (EC 1.1.1.237) followed by 3-(4-hydroxyphenyl) lactate hydrolase (Table 1). We compared the results of XTMS for this example with those from the FMM server. FMM was not able to find routes to produce raspberry ketone from 4-coumarate, neither for the target product or for its precursor *p*-hydroxybenzalacetone. We compared also the pathways found by FMM to produce 4-coumarate from *E. coli* metabolites, being able to identify both *trans*-cinnamate and tyrosine, but not the route that starts from hydroxyphenylpyruvate. Therefore, the advantages of using XTMS for this example are several: (i) XTMS was able to identify routes going from endogenous metabolites in the chassis to the target compounds, showing a better coverage of reactions databases and a good flexibility since the specific precursors do not need to be specified in advance; (ii) XTMS provided more alternative routes than FMM that can produce the target compound; (iii) XTMS ranks pathways in order to make easier the choice of the best pathways to implement; (iv) XTMS suggests enzyme sequence candidates for all the reactions in the pathway.

**Table 1.** Ranked alternative pathways producing raspberry ketone with pathway information (see Materials and Methods)

Rank	Total score	Gene score	Steps	Putatives	Toxicity	Yield	Gibbs	Unfavorable
1	2.534	0.545	4	0	-1.009	3.34e-04	-26.637	1
2	0.409	0.532	4	0	-1.009	1.00e-03	1.174	2
3	0.222	0.713	5	0	-1.026	3.34e-04	-2.004	2

The XTMS web server provides a convenient and powerful way of dealing with the complexity of pathway design for metabolic engineering. Its ability to formalize the design of metabolic circuits could be applied as a future area of development into other applications of metabolic design, such as sensing and regulation. The main feature, thus, of the XTMS server as a metabolic engineering tool is its ability of predicting novel pathways producing a desired compound and the scoring of the pathway viability, including its unique ability to score gene constructs in order to select the most appropriate gene sequence combination. As such, this work provides for the first time an open web server to access the Extended Metabolic Space that introduces a proposed pathway ranking that expands our previous retrosynthesis-based strategy.

## FUNDING

Genopole [ATIGE]; Pôle de Recherche et d’Enseignement Supérieur (PRES) UniverSud Paris [PROMISENG]; abSYNTH; Agence Nationale de la Recherche (ANR) [Chair of Excellence]. Source of open access funding: ANR.

*Conflict of interest statement*. None declared.
